# Experimental and modeling of solubility of *sitagliptin phosphate*, in supercritical carbon dioxide: proposing a new association model

**DOI:** 10.1038/s41598-023-44787-z

**Published:** 2023-10-16

**Authors:** Nedasadat Saadati Ardestani, Seyed Ali Sajadian, Nadia Esfandiari, Adrián Rojas, Chandrasekhar Garlapati

**Affiliations:** 1 Nanotechnology Research Center, Research Institute of Petroleum Industry (RIPI), 14857-336, Tehran, Iran; 2 Department of Chemical Engineering, Faculty of Engineering, University of Kashan, Kashan, 87317-53153 Iran; 3 South Zagros Oil and Gas Production, National Iranian Oil Company, Shiraz, 7135717991 Iran; 4 Department of Chemical Engineering, Marvdasht Branch, Islamic Azad University, Marvdasht, Iran; 5 Department of Science and Food Technology, Faculty of Technology, Packaging Innovation Center (LABEN), University of Santiago of Chile (USACH), Obispo Umaña 050, 9170201 Santiago, Chile; 6 Center for the Development of Nanoscience and Nanotechnology (CEDENNA), 9170124 Santiago, Chile; 7 Department of Chemical Engineering, Puducherry Technological University, Puducherry, 605014 India

**Keywords:** Chemical engineering, Nanomedicine

## Abstract

The solubility of an anti-hyperglycemic agent drug, (R)-4-oxo-4-[3-(trifluoromethyl)-5,6-dihydro [1,2,4] triazolo[4,3-a] pyrazin-7(8H)-yl]-1-(2,4,5-trifluorophenyl) butan-2-amine (also known as *Sitagliptin phosphate*) in supercritical carbon dioxide (scCO_2_) was determined by ananalytical and dynamic technique at different temperatures (308, 318, 328 and 338 K) and pressure (12–30 MPa) values. The measured solubilities were in the range of 3.02 × 10^–5^ to 5.17 × 10^–5^, 2.71 × 10^–5^ to 5.83 × 10^–5^, 2.39 × 10^–5^ to 6.51 × 10^–5^ and 2.07 × 10^–5^ to 6.98 × 10^−5^ in mole fraction at (308, 318, 328 and 338) K, respectively. The solubility data were correlated with existing density models and with a new association model.

## Introduction

Diabetes mellitus is a common metabolic disorder in which blood glucose levels are too high during a long period of time, which is increasing rapidly over the world and being considered one of the main threats to public health in the twenty-first century. It is predicted that by 2030, 366 million people worldwide will be affected by diabetes, of which 90% will be type IIof this disease^1^. The conventional diabetes treatmentis associated with some side effects such as weight gain, hypoglycemia, digestive problems, and gastric intolerance. For these this reasons, extensive researchhas been conducted to find novel drug delivery systems for this disease.

Sitagliptin phosphate, a dipeptidyl peptidase-4 (DPP-4) inhibitor, is one of the most effective anti-hyperglycemic agents which has been included in the list of diabetes drugs since 2006 with the FDA approval^2^.The use of sitagliptin phosphate effectively reduces the fasting glucose and glycosylated hemoglobin A1C (HbA1c) levels in type II diabetic patients^3^. However, the biological half life of this drug is short (about 3.6 h in rats), and it is eliminated quickly, implying the use of a high daily dose (prescribed as two doses of 50 mg day^−1^), which is not favorable for patients. This problem can be solved using an efficient drug delivery system with aprolonged and controlled release rate and increased adsorption efficiency of the drug^2, 4, 5^.

It has been reported that nano-sized drug delivery systems can deliver the required concentration of the drug to the target site of the body in a reasonable time and, as a result, increase the drug bioavailability and decrease its dosage and side effects^6^. Nanoparticles and the various types of polymericnano formulations are the most commondrug delivery systems which have shown satisfactory results in this regard. However, the method of producing these systems significantly influences their characteristics and, thus,their therapeutic efficacy. The conventional methods of producing drug delivery systems have some disadvantages, including non-uniformity of the size and morphology of the obtained particles, the consumption of a lot of organic solvent and subsequently the need forseveral purification steps to remove residual solvents and to reachpharmaceutical standards, involving the damage of the pharmaceutical compound due to severe operational conditions^7^. Therefore, the design and development of novel techniques to produce efficient drug delivery systems is one of the most attractive research areas.

It has been shown that the scCO_2_-based methods can satisfactorily replace the conventional methods used to produce various pharmaceutical formulations. The FDA’s approval for the use of CO_2_ as a permitted solvent in the pharmaceutical industry, as well as its unique characteristics, such as abundance, low cost, environmentally friendly nature, and recyclability, are the most important positive features of CO_2_^7^, which have its use in the pharmaceutical industries. Different techniques based on supercritical have been developed to produce nanosized drugs, like supercritical antisolvent (SAS)^8^, supercritical solvent impregnation (SSI)^9^, rapid expansion of a supercritical solution (RESS)^10^, and some others^11–15^.

The solubility of the desired medicine in scCO_2_ is an important factor that should be known to select the appropriate supercritical fluid-basedmethod for drug designing. For this reason, determining the solubility of different drugs in scCO_2_ has become a relevant research topic in recent years. Moreover, the theoretical determination of the solubility of drugs in scCO_2_ has attracted much attention due to the complexity and high cost of the experimental process. The empirical models, thermodynamic models based on various equations of state, intelligence models (e.g., artificial neural networks), molecular modeling and machine learning models are the common models used for this purpose. The empirical models and the equations of state-based models are the most widely used ones. The empirical models, also known as density-based models, are the simplest theoretical models that have been used to correlate the experimental solubility data of solutes in scCO_2_since 1978^16^. These models allow to calculate with acceptable accuracy the solubility of a solute based on the density of the supercritical solvent (scCO_2_) at the desired temperature and pressure, without the need of the thermodynamic and chemical properties of the solute. The of these models to predict the supercritical solubility of various drugs has been confirmed by many researchers^17–21^.

In this study, the solubility of sitagliptin phosphate in scCO_2_ was measured at temperatures of 308, 318, 328, and 338 K, and at pressures of 12–30 MPa. In addition, the solubility of the drug was theoretically determined using some well-known empirical models and a new association model.

## Experimental

### Materials used

The physicochemical properties of *sitagliptin phosphate* and the other chemicals that were utilized in the study can be found in Table 1. This includes the chemical's structureas well as its molar mass (M_w_), formula, purity, and CAS number. These substances did not require any further processing of any kind before theiruse.

**Table 1:** The molecular structure and physicochemical properties of the materials examined in this study.

Compound	Structure	*M W* (g mol^−1^)	λ_max_(nm)	CAS number	Minimum purity	Manufacture
*Sitgliptin phosphate*		407.31	268	654671-78-0	99% (m/m)	Amin Pharmaceutical Company (Iran) (Esfahan, Iran)
Carbon dioxide		44.01		124-38-9	99.99% (GC)	Aboughadare Co. (Shiraz, Iran)
Methanol		32.04		67-68-5	99% (GC)	MercK Group (Darmstadt, Germany)

### Experimental section

**Figure 1:**
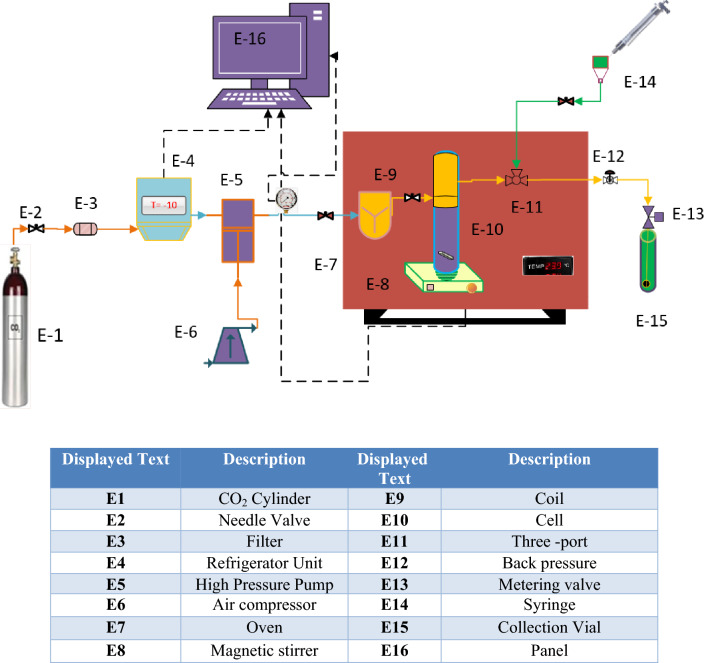
Schematic diagram of solubility device.

The Fig. 1 shows the apparatususedfor themeasurement of the Sitagliptin solubility in scCO_2_. More details about this system can be found in our previous studies^22–24^. This laboratory setup includes a CO_2_ tank, a cooling unit, a high-pressure pump, an equilibrium cell and a magnetic stirrer, which are clearly marked in the Fig. 1.

In this solubility measurement method, initially, CO_2_ from the tank at 60 bar, after passing through the molecular sieve filter (1 µm pores) and removing pollution, entered the refrigerator unit forits liquefaction by decreasingtemperature from ambient to about − 20 °C. Then liquid CO_2_ was pressurized using a high-pressure pump until reached the appropriate pressure. The pressure values were controlled and recorded on both the pressure gauge (WIKA, Germany) and pressure transmitter with an accuracy of u(P) = 0.1 MPa. After adjusting the pressure, the liquefied CO_2_ entered to the equilibrium cell whose volume was 70 mL. In the cell, liquefied CO_2_ was contacted with the drug (Sitagliptin phosphate) that was already loaded in the cell.

The equilibrium cell was placed in an oven with temperature control with an accuracy of u(T) = ± 1 K. Also, a magnet stirrer was used to achieve a complete saturation of the drug inscCO_2_. The time required for the process was 120 min. After equilibrium, using the opening a 2-status 3-way port valve and reducing the pressure,saturated scCO_2_ (600 μL)was delivered into the injection loop. Finally, by opening the micrometer valve, saturated scCO_2_was collectedinto a vial which was already loaded with 4 mL of methanol. Further, the loopwas washed with 1 mL of methanol through an external line. During the experimentspressurewas controlled with a back pressure valve. Sitagliptin absorbance in methanol was measured with a spectrophotometer- Perkin-Elmer UV–Vis at 268 nm (λ_max_) with the calibration curve (with a regression coefficient of 0.998). The experimental runs were performed three times to determine averages. The relationships used to calculate the solubility of Sitagliptin in scCO_2_ at different temperatures and pressures, in terms of mole fraction (y) and equilibrium solubility (S (g L^−1^)), are reported in our previous work^25^.

## Results and discussion

### Experimental solubility

The reliability of the solubility device was tested by determining the solubility of naphthalene at a temperature of 308 K anddifferent pressure values was measured by the used setup used in the work and the obtained data was compared with reported data by Iwai et al.^26^, Yamini et al.^27^ and Sodeifian et al.^28^. These data are listed in Table 2.

**Table 2:** Experimental solubility data of naphthalene in sc-CO_2_ at 308 K and comparison with the literature data.

Pressure (MPa)^a^	Iwai et al.^36^ (y × 10^3^)	Yamini et al.^37^ (y × 10^3^)	Sodeifian et al.^38^ (y × 10^3^)	This work (y × 10^3^)^a^
10.7	–	11.6	11.4	11.7 ± 0.2
13.8	14.1	15.2	14.3	14.8 ± 0.3
16.8	16.5	16.2	16.6	16.1 ± 0.3
20.4	17.6	17.4	17.7	17.9 ± 0.2
24.0	–	–	–	19.9 ± 0.4

^a^Standard uncertainty u are u(P) = 0.1 MPa and relative uncertainty (u_r_), u (y) = 0.10.

**Figure 2:**
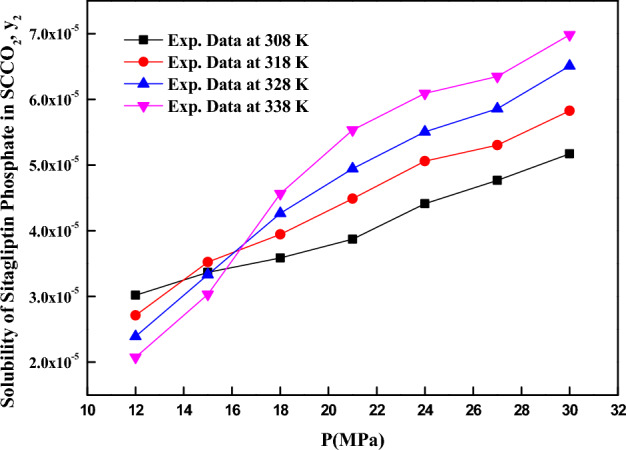
Sitagliptin phosphate solubility vs. pressure.

The solubility data for (R)-4-oxo-4-[3-(trifluoromethyl)-5,6-dihydro [1,2,4] triazolo[4,3-a] pyrazin-7(8H)-yl]-1-(2,4,5-trifluorophenyl) butan-2-amine (also known as *sitagliptin phosphate*) in scCO_2_ at different temperature (308, 318, 328 and 338 K) and pressure (12 and 30 MPa) values are shown in Table 3. Crossover points in Fig. 2 are observed for different isotherms between 15 and 16.5 MPa. Below the crossover point’s solubility increase is influenced due to increase in the density of scCO_2_, on the other hand above the crossover points the increase in solubility influenced by increase in sublimation pressure of the solute.

**Table 3:** The experimental data of sitagliptin phosphate solubility in SC-CO2 based on distinct conditions (temperatures (T) and pressures (P) for binary system).

Temperature^a^ (K)	Pressure^a^ (MPa)	Density^b^ (kg m^−3^)	y × 10^4^ (mole fraction)	Standard deviation × (10^5^)	Expanded uncertainty of mole fraction (10^4^ U)	S (Solubility (g l^−1^))
308	12	768.42	0.302	0.040	0.101	0.267
	15	816.06	0.337	0.056	0.131	0.316
	18	848.87	0.359	0.072	0.162	0.350
	21	874.4	0.387	0.090	0.197	0.389
	24	895.54	0.441	0.118	0.253	0.454
	27	913.69	0.477	0.154	0.323	0.500
	30	929.68	0.517	0.172	0.360	0.552
318	12	659.73	0.271	0.036	0.091	0.206
	15	743.17	0.352	0.059	0.138	0.301
	18	790.18	0.395	0.079	0.177	0.358
	21	823.7	0.449	0.145	0.304	0.424
	24	850.1	0.506	0.067	0.169	0.494
	27	872.04	0.530	0.100	0.227	0.531
	30	890.92	0.583	0.117	0.263	0.596
328	12	506.85	0.239	0.056	0.122	0.139
	15	654.94	0.333	0.070	0.156	0.250
	18	724.13	0.426	0.085	0.191	0.355
	21	768.74	0.494	0.137	0.292	0.436
	24	801.92	0.551	0.147	0.315	0.507
	27	828.51	0.586	0.176	0.372	0.557
	30	850.83	0.651	0.217	0.454	0.636
338	12	384.17	0.207	0.028	0.070	0.092
	15	555.23	0.303	0.051	0.119	0.193
	18	651.18	0.456	0.091	0.205	0.341
	21	709.69	0.553	0.074	0.186	0.451
	24	751.17	0.609	0.102	0.239	0.525
	27	783.29	0.635	0.127	0.285	0.571
	30	809.58	0.698	0.163	0.356	0.649

^a^Standard uncertainty u are u(T) =  ± 0.1 K; u(p) =  ± 1 bar. Also, relative standard uncertainties are obtained below 5% for mole fractions and solubilities. The value of the coverage factor k = 2 was chosen on the basis of the level of confidence of approximately 95 percent.

^b^Data from the Span–Wagner equation of state^62^.

From Fig. 2, for an isotherm higher solubility is observed at higher pressures and it is due to enhancement of scCO_2_ density at higher pressures. The effect of temperature on solubility is typical in nature and crossover points are observed. Below the crossover point’s density of scCO_2_ influence the solubility, when density of scCO_2_ is higher correspondingly solubility is high even though temperature is lower. On the other hand above the crossover point’s solubility increases with temperature and it is due to increase in sublimation pressure of the solute. Thus, temperature has duel effect on solubility via solvent density and solute sublimation pressure.

### Modeling

Solubility of drugs in supercritical fluids was modeled using different approaches that can beclassified indensity, equations of state, solid–liquid equilibrium andintelligence-based models. However, each approach has its own advantages and drawbacks. The density-based models are simple and easy to apply for data correlation due to doesn’t require solute information such as critical properties, acentric factor and sublimation pressure. *Sitagliptin phosphate* is a typical compound, and its acentric factor, critical properties (Tc and Pc) molar volumes and sublimation pressures can’t be predicted using regular group contribution methods^29–32^, due to the presence of phosphate in its chemical structure. Therefore, equation of sate (EOS) and solid–liquid equilibrium methods cannot be applied to correlate the solubility data of sitagliptin phosphate. Thus, only semi-empirical models (*i.e.,* density-based model) are useful in data correlation. In this work for data correlation Josef Chrastil model^33^, Reformulated Chrastil model^34^,Méndez-Santiago and Teja (MST) model^35^, Bartle et al. model^36^ and Kumar and Johnston (KJ) model^37^ were used. For a better data correlation, a new association model requiring only density, pressure, and temperature of scCO_2_ was proposed. Following subsections discuss about the models considered in detail.

#### Josef Chrastil model

According to this model, the solubility of solutes in SCF is expressed with the following relation:

1 $${c \mathord{\left/ {\vphantom {c {kg \cdot m^{ - 3} }}} \right. \kern-0pt} {kg \cdot m^{ - 3} }} = \left( {{{\rho_{1} } \mathord{\left/ {\vphantom {{\rho_{1} } {kg \cdot m^{ - 3} }}} \right. \kern-0pt} {kg \cdot m^{ - 3} }}} \right)^{\kappa } \exp \left( {A_{1} + {\raise0.7ex\hbox{${B_{1} }$} \!\mathord{\left/ {\vphantom {{B_{1} } {{T \mathord{\left/ {\vphantom {T K}} \right. \kern-0pt} K}}}}\right.\kern-0pt} \!\lower0.7ex\hbox{${{T \mathord{\left/ {\vphantom {T K}} \right. \kern-0pt} K}}$}}} \right)$$

where $\kappa$, $A_{1}$ and $B_{1}$ are model constants.

Equation (1) can be rearranged to mole fraction as follows^38^

2 $$\frac{{{c \mathord{\left/ {\vphantom {c {kg \cdot m^{ - 3} }}} \right. \kern-0pt} {kg \cdot m^{ - 3} }}}}{{{{\rho_{1} } \mathord{\left/ {\vphantom {{\rho_{1} } {kg \cdot m^{ - 3} }}} \right. \kern-0pt} {kg \cdot m^{ - 3} }}}}\frac{{M_{ScF} }}{{M{}_{Solute}}} = \frac{{M_{ScF} }}{{M{}_{Solute}}}\left( {{{\rho_{1} } \mathord{\left/ {\vphantom {{\rho_{1} } {kg \cdot m^{ - 3} }}} \right. \kern-0pt} {kg \cdot m^{ - 3} }}} \right)^{\kappa - 1} \exp \left( {A_{1} + {\raise0.7ex\hbox{${B_{1} }$} \!\mathord{\left/ {\vphantom {{B_{1} } {{T \mathord{\left/ {\vphantom {T K}} \right. \kern-0pt} K}}}}\right.\kern-0pt} \!\lower0.7ex\hbox{${{T \mathord{\left/ {\vphantom {T K}} \right. \kern-0pt} K}}$}}} \right)$$

3 $$mole\;ratio = \frac{{{c \mathord{\left/ {\vphantom {c {kgmol \cdot m^{ - 3} }}} \right. \kern-0pt} {kgmol \cdot m^{ - 3} }}}}{{{{\rho_{1} } \mathord{\left/ {\vphantom {{\rho_{1} } {kgmol \cdot m^{ - 3} }}} \right. \kern-0pt} {kgmol \cdot m^{ - 3} }}}} = \frac{{M_{ScF} }}{{M{}_{Solute}}}\left( {{{\rho_{1} } \mathord{\left/ {\vphantom {{\rho_{1} } {kg \cdot m^{ - 3} }}} \right. \kern-0pt} {kg \cdot m^{ - 3} }}} \right)^{\kappa - 1} \exp \left( {A_{1} + {\raise0.7ex\hbox{${B_{1} }$} \!\mathord{\left/ {\vphantom {{B_{1} } {{T \mathord{\left/ {\vphantom {T K}} \right. \kern-0pt} K}}}}\right.\kern-0pt} \!\lower0.7ex\hbox{${{T \mathord{\left/ {\vphantom {T K}} \right. \kern-0pt} K}}$}}} \right)$$

Mole fraction ($y_{2}$) and mole ratio are related as follows

4 $$\frac{{{c \mathord{\left/ {\vphantom {c {kgmol \cdot m^{ - 3} }}} \right. \kern-0pt} {kgmol \cdot m^{ - 3} }}}}{{{{\rho_{1} } \mathord{\left/ {\vphantom {{\rho_{1} } {kgmol \cdot m^{ - 3} }}} \right. \kern-0pt} {kgmol \cdot m^{ - 3} }}}} = \frac{{{{y_{2} } \mathord{\left/ {\vphantom {{y_{2} } {mole\;fraction}}} \right. \kern-0pt} {mole\;fraction}}}}{{1 - {{y_{2} } \mathord{\left/ {\vphantom {{y_{2} } {mole\;fraction}}} \right. \kern-0pt} {mole\;fraction}}}}$$

5 $${{y_{2} } \mathord{\left/ {\vphantom {{y_{2} } {mole\;fraction}}} \right. \kern-0pt} {mole\;fraction}} = {{\frac{{{c \mathord{\left/ {\vphantom {c {kgmol \cdot m^{ - 3} }}} \right. \kern-0pt} {kgmol \cdot m^{ - 3} }}}}{{{{\rho_{1} } \mathord{\left/ {\vphantom {{\rho_{1} } {kgmol \cdot m^{ - 3} }}} \right. \kern-0pt} {kgmol \cdot m^{ - 3} }}}}} \mathord{\left/ {\vphantom {{\frac{{{c \mathord{\left/ {\vphantom {c {kgmol \cdot m^{ - 3} }}} \right. \kern-0pt} {kgmol \cdot m^{ - 3} }}}}{{{{\rho_{1} } \mathord{\left/ {\vphantom {{\rho_{1} } {kgmol \cdot m^{ - 3} }}} \right. \kern-0pt} {kgmol \cdot m^{ - 3} }}}}} {\left[ {1 + \frac{{{c \mathord{\left/ {\vphantom {c {kgmol \cdot m^{ - 3} }}} \right. \kern-0pt} {kgmol \cdot m^{ - 3} }}}}{{{{\rho_{1} } \mathord{\left/ {\vphantom {{\rho_{1} } {kgmol \cdot m^{ - 3} }}} \right. \kern-0pt} {kgmol \cdot m^{ - 3} }}}}} \right]}}} \right. \kern-0pt} {\left[ {1 + \frac{{{c \mathord{\left/ {\vphantom {c {kgmol \cdot m^{ - 3} }}} \right. \kern-0pt} {kgmol \cdot m^{ - 3} }}}}{{{{\rho_{1} } \mathord{\left/ {\vphantom {{\rho_{1} } {kgmol \cdot m^{ - 3} }}} \right. \kern-0pt} {kgmol \cdot m^{ - 3} }}}}} \right]}}$$

6 $${{y_{2} } \mathord{\left/ {\vphantom {{y_{2} } {mole\;fraction}}} \right. \kern-0pt} {mole\;fraction}} = {{\frac{{M_{ScF} }}{{M{}_{Solute}}}\left( {{{\rho_{1} } \mathord{\left/ {\vphantom {{\rho_{1} } {kg \cdot m^{ - 3} }}} \right. \kern-0pt} {kg \cdot m^{ - 3} }}} \right)^{\kappa - 1} \exp \left( {A_{1} + {\raise0.7ex\hbox{${B_{1} }$} \!\mathord{\left/ {\vphantom {{B_{1} } {{T \mathord{\left/ {\vphantom {T K}} \right. \kern-0pt} K}}}}\right.\kern-0pt} \!\lower0.7ex\hbox{${{T \mathord{\left/ {\vphantom {T K}} \right. \kern-0pt} K}}$}}} \right)} \mathord{\left/ {\vphantom {{\frac{{M_{ScF} }}{{M{}_{Solute}}}\left( {{{\rho_{1} } \mathord{\left/ {\vphantom {{\rho_{1} } {kg \cdot m^{ - 3} }}} \right. \kern-0pt} {kg \cdot m^{ - 3} }}} \right)^{\kappa - 1} \exp \left( {A_{1} + {\raise0.7ex\hbox{${B_{1} }$} \!\mathord{\left/ {\vphantom {{B_{1} } {{T \mathord{\left/ {\vphantom {T K}} \right. \kern-0pt} K}}}}\right.\kern-0pt} \!\lower0.7ex\hbox{${{T \mathord{\left/ {\vphantom {T K}} \right. \kern-0pt} K}}$}}} \right)} {\left[ {1 + \frac{{M_{ScF} }}{{M{}_{Solute}}}\left( {{{\rho_{1} } \mathord{\left/ {\vphantom {{\rho_{1} } {kg \cdot m^{ - 3} }}} \right. \kern-0pt} {kg \cdot m^{ - 3} }}} \right)^{\kappa - 1} \exp \left( {A_{1} + {\raise0.7ex\hbox{${B_{1} }$} \!\mathord{\left/ {\vphantom {{B_{1} } {{T \mathord{\left/ {\vphantom {T K}} \right. \kern-0pt} K}}}}\right.\kern-0pt} \!\lower0.7ex\hbox{${{T \mathord{\left/ {\vphantom {T K}} \right. \kern-0pt} K}}$}}} \right)} \right]}}} \right. \kern-0pt} {\left[ {1 + \frac{{M_{ScF} }}{{M{}_{Solute}}}\left( {{{\rho_{1} } \mathord{\left/ {\vphantom {{\rho_{1} } {kg \cdot m^{ - 3} }}} \right. \kern-0pt} {kg \cdot m^{ - 3} }}} \right)^{\kappa - 1} \exp \left( {A_{1} + {\raise0.7ex\hbox{${B_{1} }$} \!\mathord{\left/ {\vphantom {{B_{1} } {{T \mathord{\left/ {\vphantom {T K}} \right. \kern-0pt} K}}}}\right.\kern-0pt} \!\lower0.7ex\hbox{${{T \mathord{\left/ {\vphantom {T K}} \right. \kern-0pt} K}}$}}} \right)} \right]}}$$

**Figure 3:**
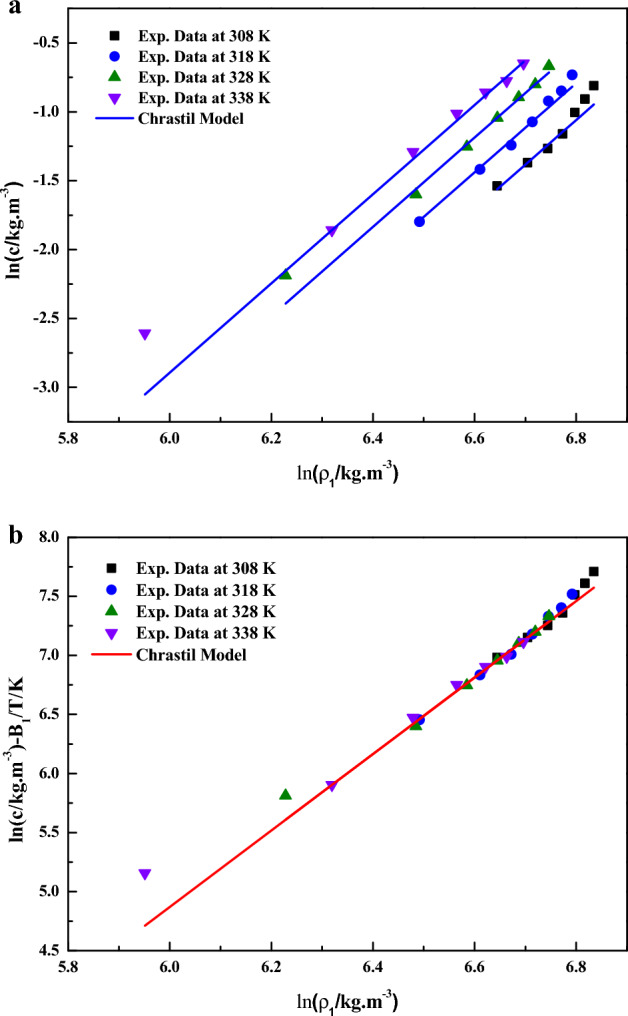
(**a**) Experimental data of supercritical solubility of sitagliptin phosphate (points) compared to data calculated with the Chrastil model (line). (**b**) Results of self-consistency analysis for the Chrastil model.

In Eq. (6), the model constants are treated as independent of temperature and their values are obtained by regression with experimental data^38^. The obtained values are reported in Table 4. It is quite evident that a linear plot is observed when the data is depicted as $\ln \left( {{c \mathord{\left/ {\vphantom {c {kg \cdot m^{ - 3} }}} \right. \kern-0pt} {kg \cdot m^{ - 3} }}} \right)$ vs. $\ln \left( {{{\rho_{1} } \mathord{\left/ {\vphantom {{\rho_{1} } {kg \cdot m^{ - 3} }}} \right. \kern-0pt} {kg \cdot m^{ - 3} }}} \right)$ (Fig. 3a) and as $\ln \left( {{c \mathord{\left/ {\vphantom {c {kg \cdot m^{ - 3} }}} \right. \kern-0pt} {kg \cdot m^{ - 3} }}} \right) - B_{1} /T/K$ vs. $\ln \left( {{{\rho_{1} } \mathord{\left/ {\vphantom {{\rho_{1} } {kg \cdot m^{ - 3} }}} \right. \kern-0pt} {kg \cdot m^{ - 3} }}} \right)$ (Fig. 3b), this confirms the applicability of the Chrastil model to the solubility data^39^. From the constant $B_{1}$, total heat of reaction is calculated (i.e., $\Delta H_{Total} = B_{1} R$), the obtained values are reported in Table 5

**Table 4:** The correlation results of the sitagliptin phosphate–CO_2_ system provided by semi-empirical models and new association model.

Sl. no	Name of the model & Equation number	Model parameters	R^2^	R^2^_adj_	AARD%
1	Chrastil Model & Eq. (6)	$\kappa =$ 3.2364; $A_{1} =$− 14.548; $B_{1} =$− 2624.0	0.92249	0.91951	4.97
2	Reformulated Chrastil Model & Eq. (7)	$\kappa^{\prime} =$ 3.2439; $A_{2} =$− 17.896; $B_{2} =$− 1910.2	0.96697	0.96570	4.95
3	MT Model & Eq. (8)	$A_{3} =$− 6486.6; $B_{3} =$ 2.193; $C_{3} =$ 10.103	0.91635	0.9059	8.721
4	Bartle et. al. Model & Eq. (9)	$A_{4} =$ 9.8241; $B_{4} =$− 4861,2 $C_{4} =$ 0.0065735	0.89618	0.89219	9.06
5	KJ Model & Eq. (10)	$A_{5} =$− 4.197 $B_{5} =$ 0.0031559 $C_{5} =$− 2671.2	0.98536	0.98480	3.16
6	New Association Model & Eq. (30)	$\kappa^{\prime\prime} =$ 1.1945; $A_{6} =$− 1519.5; $B_{6} =$ 2.1846; $C_{6} =$ 0.0024843; $D_{6} =$− 20.894	0.98817	0.98772	2.53

**Table 5:** Calculated enthalpies of sitagliptin phosphate–CO_2_ system provided by semi-empirical models.

Model	Property		
	Total enthalpy in kJ mol^−1^	Sublimation enthalpy, kJ mol^−1^	Solvation enthalpy, kJ mol^−1^
Chrastil Model^a^	21.815^a^		*− 18.601^b^
Reformulated Chrastil Model^c^	15.881^c^		**− 24.535^d^
Bartle et al. Model^e^		40.416^e^	

*Solvation enthalpy^b^ = Total Enthalpy obtained from Chrastil Model^a^-Sublimation Enthalpy obtained from Bartle et al. Model^e^; **Solvation enthalpy^d^ = Total Enthalpy obtained from reformulated Chrastil Model^c^-Sublimation Enthalpy obtained from Bartle et al. Model^e^; A negative sign is attributed solvation enthalpy.

#### Reformulated Chrastil model

According to this model, solubility is a function of $\kappa^{\prime}$(association number), $\rho_{1}$ (solvent density (scCO_2_)) and T (temperature). In Eq. (7), it is important to note that depending on the reference fugacity units, R units are selected.

7 $${{y_{2} } \mathord{\left/ {\vphantom {{y_{2} } {mole\;fraction}}} \right. \kern-0pt} {mole\;fraction}} = \left( {\frac{{{R \mathord{\left/ {\vphantom {R {atm \cdot m^{3} \cdot kgmol^{ - 1} K^{ - 1} }}} \right. \kern-0pt} {atm \cdot m^{3} \cdot kgmol^{ - 1} K^{ - 1} }}{T \mathord{\left/ {\vphantom {T K}} \right. \kern-0pt} K}{{\rho_{1} } \mathord{\left/ {\vphantom {{\rho_{1} } {kg \cdot m^{ - 3} }}} \right. \kern-0pt} {kg \cdot m^{ - 3} }}}}{{{{M_{ScF} } \mathord{\left/ {\vphantom {{M_{ScF} } {kg \cdot kgmol^{ - 1} }}} \right. \kern-0pt} {kg \cdot kgmol^{ - 1} }} \cdot {{f^{ \cdot } } \mathord{\left/ {\vphantom {{f^{ \cdot } } {1\;atm}}} \right. \kern-0pt} {1\;atm}}}}} \right)^{{\kappa^{\prime} - 1}} \exp \left( {A_{2} + \frac{{B_{2} }}{{{T \mathord{\left/ {\vphantom {T K}} \right. \kern-0pt} K}}}} \right)$$

where R denotes universal gas constant (0.082057 atm m^3^ kgmole^−1^ K^−1^), $M_{ScF}$ is molecular weight of solvent (For CO_2_ 44.01 kg kgmol^−1^), $f^{ * }$ is reference fugacity (1 atm) and $A_{2} \;$ and $\;B_{2}$ are the reformulated model constants.

**Figure 4:**
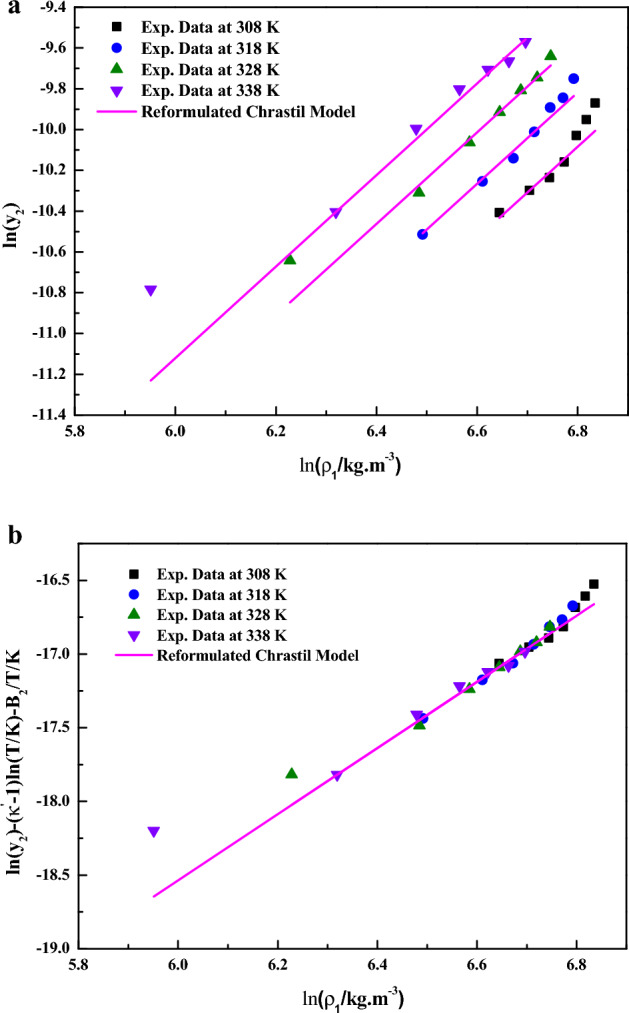
(**a**) Experimental data of supercritical solubility of sitagliptin phosphate (points) compared to data calculated with the Reformulated Chrastil Model (line). (**b**) Results of self-consistency analysis for the Reformulated Chrastil Model.

In Eq. (7), the model constants were treated as independent of temperature and their values were obtained by regression with experimental data. The obtained values are reported in Table 4 It is quite evident that a linear plot is observed when the data are depicted as $\ln \left( {y_{2} } \right)$ vs. $\ln \left( {{{\rho_{1} } \mathord{\left/ {\vphantom {{\rho_{1} } {kg \cdot m^{ - 3} }}} \right. \kern-0pt} {kg \cdot m^{ - 3} }}} \right)$ (Fig. 4a) and as $\ln \left( {y_{2} } \right) - (\kappa^{\prime} - 1)\ln \left( T \right) - B_{2} /T/K$ vs. ${{\ln \;(\rho_{1} } \mathord{\left/ {\vphantom {{\ln \;(\rho_{1} } {kg \cdot m^{ - 3} }}} \right. \kern-0pt} {kg \cdot m^{ - 3} }})$ (Fig. 4b), this confirms the applicability of the reformulated Chrastil model to the solubility data. From the constant $B_{2}$, total heat of reaction is calculated (i.e.,$\Delta H_{Total} = B_{2} R$), the obtained values are reported in Table 5.

#### Méndez-Santiago and Teja (MT) model

Internal consistency of the measured solubility data was checked with this model. It is stated as Eq. (8) and when $T\ln \left( {y_{2} P} \right) - C_{3} T$ vs. $\rho_{1}$ is established, all data points fall around a single straight line.

8 $${T \mathord{\left/ {\vphantom {T {K \cdot \ln \left( {y_{2} {P \mathord{\left/ {\vphantom {P {bar}}} \right. \kern-0pt} {bar}}} \right)}}} \right. \kern-0pt} {K \cdot \ln \left( {y_{2} {P \mathord{\left/ {\vphantom {P {bar}}} \right. \kern-0pt} {bar}}} \right)}} = A_{3} + B_{3} \cdot {{\rho_{1} } \mathord{\left/ {\vphantom {{\rho_{1} } {kg \cdot m^{ - 3} + C_{3} \cdot {T \mathord{\left/ {\vphantom {T K}} \right. \kern-0pt} K}}}} \right. \kern-0pt} {kg \cdot m^{ - 3} + C_{3} \cdot {T \mathord{\left/ {\vphantom {T K}} \right. \kern-0pt} K}}}$$

where *A*_*3*_ to *C*_*3*_ are the model constants.

**Figure 5:**
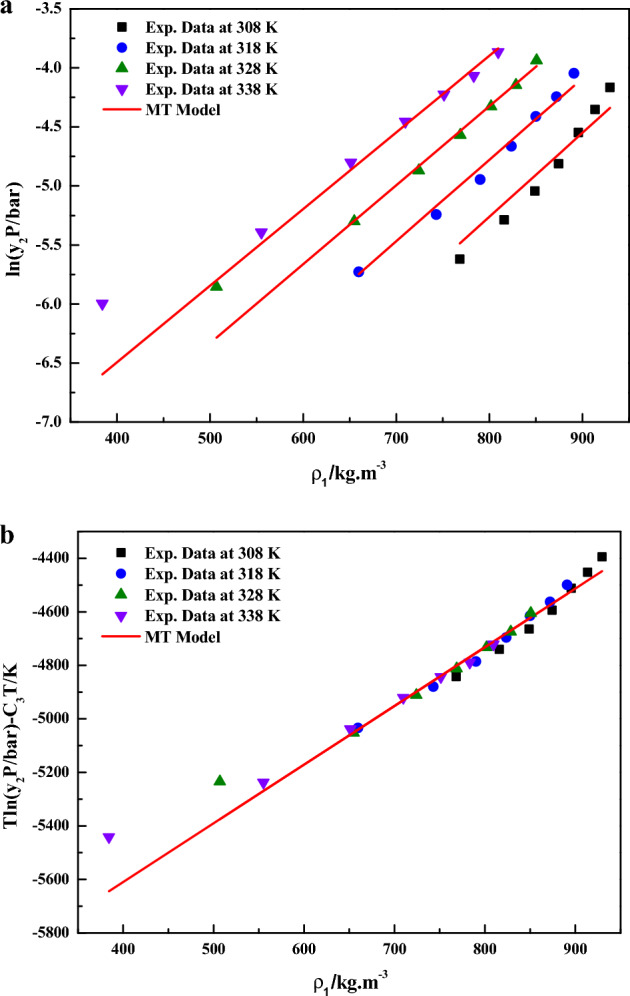
(**a**) Experimental data of supercritical solubility of sitagliptin phosphate (points) compared to data calculated with the MT Model (line). (**b**) Results of self-consistency analysis for the MT Model.

In Eq. (8), the model constants were treated as independent of temperature and their values were obtained by regression with experimental data. The obtained values were reported in Table 4. The experimental data obtained in this work is checked for consistency with the help of Mendez-Santiago and Teja model (MT model). According to the MT model, the solubility data at different temperatures collapsed into a single line. It is quite evident that linear plots are observed when the data are depicted as $\ln \left( {y_{2} \cdot P/bar} \right)$ vs. ${{\rho_{1} } \mathord{\left/ {\vphantom {{\rho_{1} } {kg \cdot m^{ - 3} }}} \right. \kern-0pt} {kg \cdot m^{ - 3} }}$ (Fig. 5a) and as ${T \mathord{\left/ {\vphantom {T {K\ln \left( {y_{2} \cdot P/bar} \right)}}} \right. \kern-0pt} {K\ln \left( {y_{2} \cdot P/bar} \right)}} - C_{3} T/K$ vs. ${{\rho_{1} } \mathord{\left/ {\vphantom {{\rho_{1} } {kg \cdot m^{ - 3} }}} \right. \kern-0pt} {kg \cdot m^{ - 3} }}$(Fig. 5b), this confirms the applicability of the MT model to the solubility data.

#### Bartle et al., model

According to the model the solubility was expressed as Eq. (9)

9 $$\ln \left( {\frac{{y_{2} \cdot P}}{{P_{ref} }}} \right) = A_{4} + \frac{{B_{4} }}{{{T \mathord{\left/ {\vphantom {T K}} \right. \kern-0pt} K}}} + C_{4} \left( {{{\rho_{1} } \mathord{\left/ {\vphantom {{\rho_{1} } {kg \cdot m^{ - 3} }}} \right. \kern-0pt} {kg \cdot m^{ - 3} }} - {{\rho_{ref} } \mathord{\left/ {\vphantom {{\rho_{ref} } {kg \cdot m^{ - 3} }}} \right. \kern-0pt} {kg \cdot m^{ - 3} }}} \right)$$

where reference pressure is 0.1 MPa or 1 bar, reference density is 700 kg m^−3^ and *A*_*4*_ to *C*_*4 *_are the model constants. From the constant $B_{4}$, sublimation enthalpy is calculated (i.e.,$\Delta_{sub} H = - B_{4} R$).

**Figure 6:**
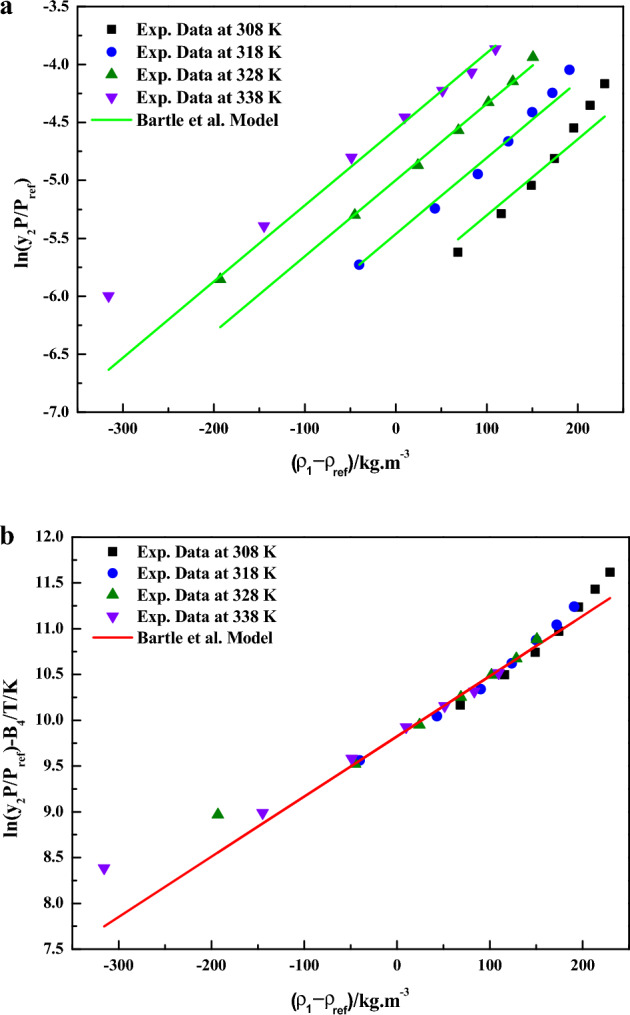
(**a**) Experimental data of supercritical solubility of sitagliptin phosphate (points) compared to data calculated with the Bartle et al. Model (line). (**b**) Results of self-consistency analysis for the Bartle et al. Model.

In Eq. (9), the model constants were treated as independent of temperature and their values were obtained by regression with experimental data. The obtained values were reported in Table 4 it is quite evident that linear plots are observed when the data are depicted as $\ln \left( {\frac{{y_{2} P}}{{P_{ref} }}} \right)$ vs. ${{\left( {\rho_{1} - \rho_{ref} } \right)} \mathord{\left/ {\vphantom {{\left( {\rho_{1} - \rho_{ref} } \right)} {kg \cdot m^{ - 3} }}} \right. \kern-0pt} {kg \cdot m^{ - 3} }}$(Fig. 6a) and as $\ln \left( {\frac{{y_{2} P}}{{P_{ref} }}} \right) - \frac{{B_{4} }}{{{T \mathord{\left/ {\vphantom {T K}} \right. \kern-0pt} K}}}$ vs. ${{\left( {\rho_{1} - \rho_{ref} } \right)} \mathord{\left/ {\vphantom {{\left( {\rho_{1} - \rho_{ref} } \right)} {kg \cdot m^{ - 3} }}} \right. \kern-0pt} {kg \cdot m^{ - 3} }}$ (Fig. 6b) this confirms the applicability of the Bartle et al. model to the solubility data.

#### Kumar and Johnston (KJ) model

According to the model the solubility was expressed as Eq. (10)

10 $$\ln \left( {y_{2} } \right) = A_{5} + B_{5} \left( {{{\rho_{1} } \mathord{\left/ {\vphantom {{\rho_{1} } {kg \cdot m^{ - 3} }}} \right. \kern-0pt} {kg \cdot m^{ - 3} }}} \right) + \frac{{C_{5} }}{{{T \mathord{\left/ {\vphantom {T K}} \right. \kern-0pt} K}}}$$

where *A*_*5*_ to *C*_*5*_ are the model constants.

**Figure 7:**
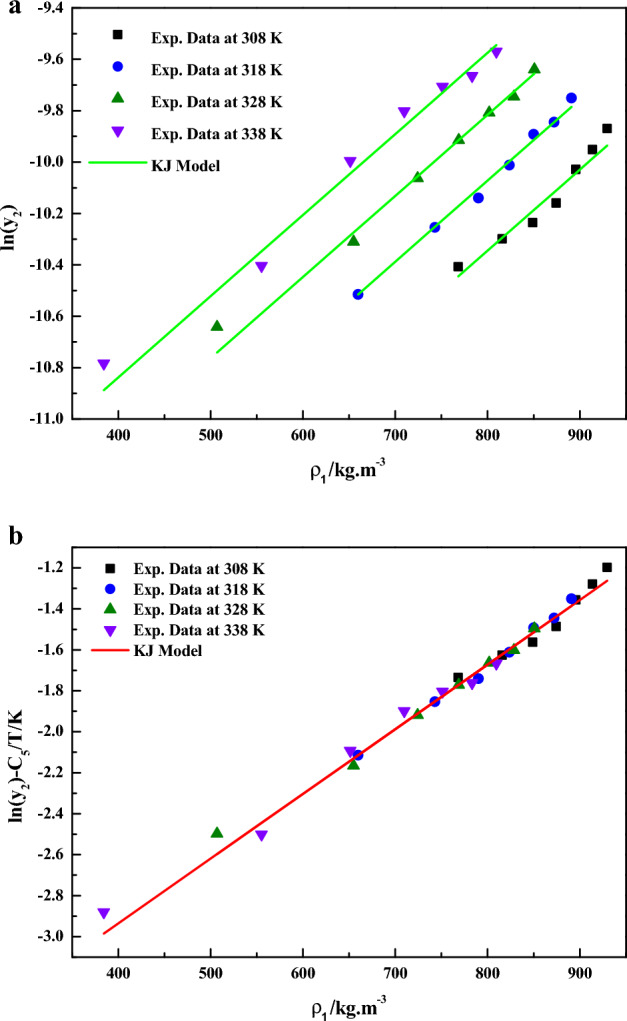
(**a**) Experimental data of supercritical solubility of sitagliptin phosphate (points) compared to data calculated with the KJ Model (line). (**b**) Results of self-consistency analysis for the KJ Model.

In Eq. (10), the model constants were treated as independent of temperature and their values were obtained by regression with experimental data. The obtained values were reported in Table 3 it is quite evident that a linear plots are observed when the data are depicted as $\ln \left( {y_{2} } \right) - \frac{{C_{5} }}{{{T \mathord{\left/ {\vphantom {T K}} \right. \kern-0pt} K}}}$ vs.${{\rho_{1} } \mathord{\left/ {\vphantom {{\rho_{1} } {kg \cdot m^{ - 3} }}} \right. \kern-0pt} {kg \cdot m^{ - 3} }}$(Fig. 7a) and as $\ln \left( {y_{2} } \right) - \frac{{C_{5} }}{{{T \mathord{\left/ {\vphantom {T K}} \right. \kern-0pt} K}}}$ vs. ${{\rho_{1} } \mathord{\left/ {\vphantom {{\rho_{1} } {kg \cdot m^{ - 3} }}} \right. \kern-0pt} {kg \cdot m^{ - 3} }}$(Fig. 7b), this confirms the applicability of the KJ model to the solubility data.

#### New association model

If one molecule of a solute (A) associates with $\kappa^{\prime\prime}$ molecules of solvent (B) to form one molecule of a solvato complex $AB_{\kappa }$ in equilibrium with the gaseous system^33^,

11 $$A + \kappa^{\prime\prime}B \Leftrightarrow AB_{\kappa }$$

Equation (12) represents the equilibrium constant in terms of the individual component’s fugacity

12 $$K_{f} = \frac{{\left( {{\raise0.7ex\hbox{${\hat{f}_{{AB_{\kappa } }} }$} \!\mathord{\left/ {\vphantom {{\hat{f}_{{AB_{\kappa } }} } {f_{{AB_{\kappa } }}^{ * } }}}\right.\kern-0pt} \!\lower0.7ex\hbox{${f_{{AB_{\kappa } }}^{ * } }$}}} \right)_{ScP} }}{{\left( {\frac{{\hat{f}_{A} }}{{f_{A}^{ * } }}} \right)_{S} \;\left( {\left( {\frac{{\hat{f}_{B} }}{{f_{B}^{ * } }}} \right)^{{\kappa^{\prime\prime}}} } \right)_{ScP} }}$$

where ScP represents the supercritical phase, S represents the solute phase and $f^{ * }$ is reference fugacity.

The fugacity for each component can be calculated by the following equations^40–42^.

13 $$\hat{f}_{A} = y_{A} \hat{\phi }_{A} P$$

14 $$\hat{f}_{B} = y_{B} \hat{\phi }_{B} P$$

15 $$\hat{f}_{{AB_{\kappa } }} = y_{{AB_{\kappa } }} \hat{\phi }_{{AB_{\kappa } }} P$$

16 $$f_{{AB_{\kappa } }}^{ * } = \phi_{{AB_{\kappa } }}^{ * } \;P^{ * }$$

17 $$f_{A}^{ * } = \phi_{A}^{ * } \;P^{ * }$$

18 $$f_{B}^{ * } = \phi_{B}^{ * } \;P^{ * }$$

Here the main assumption is fluid-phase component does not dissolve in the solid. i.e., the solid is pure. Solute A exists in an associated state in ScP

19 $$y_{B} + y_{{AB_{\kappa } }} = 1$$

where $y_{B} ,y_{{AB_{\kappa } }}$ are mole fraction of solvent and solvato complex respectively.

Since the solute A mainly exists in an associating state, the solubility of solute A in ScP is^43–45^

20 $$y_{2} = \frac{{y_{{AB_{\kappa } }} }}{{1 + \kappa^{\prime\prime}\;y_{{AB_{\kappa } }} }}$$

when standard state of the solute A is treated as pure solute at system pressure (P) and temperature (T), then

21 $$\hat{f}_{A} = f_{A}$$

The fugacity of pure solute can be written as

22 $$f_{A} = P_{A}^{sub} \exp \left( {\frac{{V_{A} (P - P_{A}^{sub} )}}{RT}} \right)$$

where $P_{A}^{sub}$ is the sublimation pressure of the pure solid, and $V_{A}$ is the molar volume of the pure solid at system temperature(*T*), and pressure, (*P*).

Substituting Eqs. (13)–(18) and Eq. (22) in Eq. (12) gives Eq. (23)

23 $$K_{f} = \frac{{\left( {\frac{{y_{{AB_{\kappa } }} \hat{\phi }_{{AB_{\kappa } }} P}}{{\phi_{{AB_{\kappa } }}^{ * } P^{ * } }}} \right)}}{{\left( {\frac{{P_{A}^{sub} \exp \left( {\frac{{V_{A} \left( {P - P_{A}^{sub} } \right)}}{RT}} \right)}}{{\phi_{A}^{ * } P^{ * } }}} \right)\;\left( {\frac{{y_{B} \hat{\phi }_{B} P}}{{\phi_{B}^{ * } P^{ * } }}} \right)^{{\kappa^{\prime\prime}}} }}$$

24 $$\begin{aligned} \ln \left( {K_{f} } \right) & = \ln \left( {y_{{AB_{\kappa } }} } \right) + \ln \left( {{\raise0.7ex\hbox{${\hat{\phi }_{{AB_{\kappa } }} }$} \!\mathord{\left/ {\vphantom {{\hat{\phi }_{{AB_{\kappa } }} } {\phi_{{AB_{\kappa } }}^{ * } }}}\right.\kern-0pt} \!\lower0.7ex\hbox{${\phi_{{AB_{\kappa } }}^{ * } }$}}} \right) + \ln \left( {{\raise0.7ex\hbox{$P$} \!\mathord{\left/ {\vphantom {P {P^{ * } }}}\right.\kern-0pt} \!\lower0.7ex\hbox{${P^{ * } }$}}} \right) + \ln \left( {\phi_{A}^{ * } } \right) - \ln \left( {{\raise0.7ex\hbox{${P_{A}^{sub} }$} \!\mathord{\left/ {\vphantom {{P_{A}^{sub} } {P^{ * } }}}\right.\kern-0pt} \!\lower0.7ex\hbox{${P^{ * } }$}}} \right) - \frac{{V_{A} \left( {P - P_{A}^{sub} } \right)}}{RT} \\ & \quad \quad - \kappa^{\prime\prime}\ln \left( {y_{B} } \right) - \kappa^{\prime\prime}\ln \left( {{\raise0.7ex\hbox{${\hat{\phi }_{B} }$} \!\mathord{\left/ {\vphantom {{\hat{\phi }_{B} } {\phi_{B}^{ * } }}}\right.\kern-0pt} \!\lower0.7ex\hbox{${\phi_{B}^{ * } }$}}} \right) - \kappa^{\prime\prime}\ln \left( {{\raise0.7ex\hbox{$P$} \!\mathord{\left/ {\vphantom {P {P^{ * } }}}\right.\kern-0pt} \!\lower0.7ex\hbox{${P^{ * } }$}}} \right) \\ \end{aligned}$$

The equilibrium constant,$K_{f} ,$ may be expressed as $\ln \left( {K_{f} } \right) = \frac{{\Delta H_{s} }}{RT} + q_{s}$.

where $$\Delta H_{s}$$, the heat of solvation and *q*_*s*_is a constant and $${\raise0.7ex\hbox{${V_{A} P}$} \!\mathord{\left/ {\vphantom {{V_{A} P} {RT}}}\right.\kern-0pt} \!\lower0.7ex\hbox{${RT}$}}$$ may be expressed as $${\raise0.7ex\hbox{${ZV_{A} \rho }$} \!\mathord{\left/ {\vphantom {{ZV_{A} \rho } M}}\right.\kern-0pt} \!\lower0.7ex\hbox{$M$}}$$ where $$\rho$$ is the density of the supercritical phase. At the supercritical state, $$\rho$$ is a function of three variables namely pressure, temperature and composition. Thus, the fugacities in Eq. (24) are a very complex function of pressure, temperature and composition.

Then Eq. (24) may be expressed as

25 $$\begin{aligned} \ln \left( {y_{{AB_{\kappa } }} } \right) - \kappa^{\prime\prime}\ln \left( {y_{B} } \right) + \left( {1 - \kappa^{\prime\prime}} \right)\ln \left( {{\raise0.7ex\hbox{$P$} \!\mathord{\left/ {\vphantom {P {P^{ * } }}}\right.\kern-0pt} \!\lower0.7ex\hbox{${P^{ * } }$}}} \right) & = - \ln \left( {{\raise0.7ex\hbox{${\hat{\phi }_{{AB_{\kappa } }} }$} \!\mathord{\left/ {\vphantom {{\hat{\phi }_{{AB_{\kappa } }} } {\phi_{{AB_{\kappa } }}^{ * } }}}\right.\kern-0pt} \!\lower0.7ex\hbox{${\phi_{{AB_{\kappa } }}^{ * } }$}}} \right) - \ln \left( {\phi_{A}^{ * } } \right) + \ln \left( {P_{A}^{sub} } \right) - \ln \left( {P^{ * } } \right) \\ & \quad \quad + \frac{{ZV_{A} \rho }}{M} - \frac{{V_{A} P_{A}^{sub} }}{RT} + \kappa^{\prime\prime}\ln \left( {{\raise0.7ex\hbox{${\hat{\phi }_{B} }$} \!\mathord{\left/ {\vphantom {{\hat{\phi }_{B} } {\phi_{B}^{ * } }}}\right.\kern-0pt} \!\lower0.7ex\hbox{${\phi_{B}^{ * } }$}}} \right) + \frac{{\Delta H_{s} }}{RT} + q_{s} \\ \end{aligned}$$

The sublimation pressure of the solid solute may be expressed as

26 $$R\ln \left( {P_{A}^{sub} } \right) = \beta + \frac{\gamma }{T} + \Delta_{sub} \delta \ln \left( {\frac{T}{298.15}} \right)$$

where $\beta$, $\gamma$ and $\Delta_{sub} \delta$ are temperature independent parameters.

When $\frac{{V_{A} P_{A}^{sub} }}{RT}$(~ 10^–9^) term is neglected (since the sublimation pressures (~ 10^–4^) and molar volume of solid solutes (~ 10^–4^) are very low) and density of solution is treated as approximately as density of supercritical fluid

27 $$\ln \left( {y_{{AB_{\kappa } }} } \right) - \kappa^{\prime\prime}\ln \left( {y_{B} } \right) + \left( {1 - \kappa^{\prime\prime}} \right)\ln \left( {{\raise0.7ex\hbox{$P$} \!\mathord{\left/ {\vphantom {P {P^{ * } }}}\right.\kern-0pt} \!\lower0.7ex\hbox{${P^{ * } }$}}} \right) = \frac{{A_{6} }}{T} + B_{6} \ln \left( T \right) + C_{6} \rho_{1} + D_{6}$$

where $$A_{6} = \frac{{\Delta H_{s} }}{R} + \frac{\gamma }{R}$$, $$B_{6} = {{\Delta_{sub} \delta } \mathord{\left/ {\vphantom {{\Delta_{sub} \delta } R}} \right. \kern-0pt} R}$$, $$C_{6} = \frac{{ZV_{A} }}{M}$$ and $$D_{6} = - \ln \left( {\frac{{\left( {\phi_{A}^{ * } } \right)\left( {{\raise0.7ex\hbox{${\hat{\phi }_{{AB_{\kappa } }} }$} \!\mathord{\left/ {\vphantom {{\hat{\phi }_{{AB_{\kappa } }} } {\phi_{{AB_{\kappa } }}^{ * } }}}\right.\kern-0pt} \!\lower0.7ex\hbox{${\phi_{{AB_{\kappa } }}^{ * } }$}}} \right)}}{{\left( {{\raise0.7ex\hbox{${\hat{\phi }_{B} }$} \!\mathord{\left/ {\vphantom {{\hat{\phi }_{B} } {\phi_{B}^{ * } }}}\right.\kern-0pt} \!\lower0.7ex\hbox{${\phi_{B}^{ * } }$}}} \right)^{\kappa } }}} \right) - \ln P^{ * } + q_{s} + \frac{\beta }{R} - \frac{{\Delta_{sub} \delta \ln \left( {298.15} \right)}}{R}$$

Equation (27) may be written as Eq. (28)

28 $$y_{{AB_{\kappa } }} = \left( {y_{B} } \right)^{{\kappa^{\prime\prime}}} \left( {\frac{P}{{P^{ * } }}} \right)^{{\left( {\kappa^{\prime\prime} - 1} \right)}} \exp \left( {\frac{{A_{6} }}{T} + B_{6} \ln (T) + C_{6} \rho_{1} + D_{6} } \right)$$

Because the solubility of drug in scCO_2_ are very dilute, therefore for a binary system we may assume $y_{B}$ is unity. Then Eq. (28) reduced to Eq. (29)

29 $$y_{{AB_{\kappa } }} = \left( {\frac{P}{{P^{ * } }}} \right)^{{\left( {\kappa^{\prime\prime} - 1} \right)}} \exp \left( {\frac{{A_{6} }}{T} + B_{6} \ln (T) + C_{6} \rho_{1} + D_{6} } \right)$$

Combining Eq. (20) and Eq. (29) gives expression for solubility Eq. (30)

30 $$y_{2} = \frac{{\left( {\frac{P}{{P^{ * } }}} \right)^{{\left( {\kappa^{\prime\prime} - 1} \right)}} \exp \left( {\frac{{A_{6} }}{T} + B_{6} \ln (T) + C_{6} \rho_{1} + D_{6} } \right)}}{{1 + \kappa^{\prime\prime}\left( {\frac{P}{{P^{ * } }}} \right)^{{\left( {\kappa^{\prime\prime} - 1} \right)}} \exp \left( {\frac{{A_{6} }}{T} + B_{6} \ln (T) + C_{6} \rho_{1} + D_{6} } \right)}}$$

**Figure 8:**
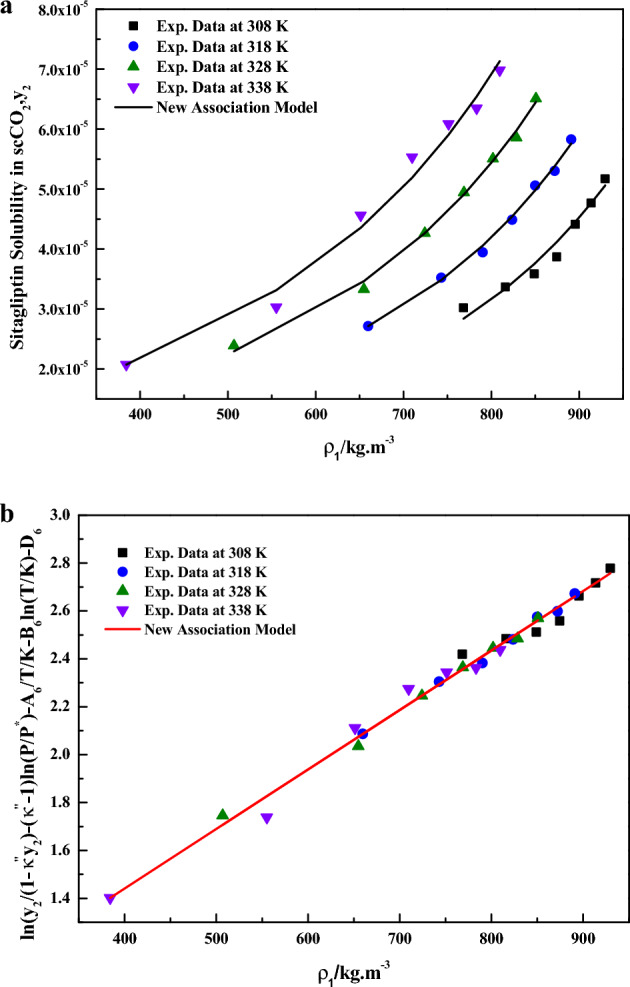
(**a**) Sitagliptin phosphate solubility,$y_{2}$ vs. $\rho_{1} /kg \cdot m^{ - 3}$. (**b**) Results of self-consistency analysis for the New Association Model.

From Eq. (30),it is clear that solubility is a function of density, temperature and association number (i.e.,$y_{2} = y_{2} (\rho_{1} ,T,\kappa^{\prime\prime})$) and further all equations are dimensionally consistent. Hereafter, this equation may be called as the new association model. In Eq. (30), the model constants were treated as independent of temperature and their values were obtained by regression with experimental data. The obtained values are reported in Table 4 it is quite evident that a better fit is observed when the data is plotted as Sitagliptin Phosphate solubility, $y_{2}$ vs. $\rho_{1} /kg \cdot m^{ - 3}$(Fig. 8a). It is important to note that Fig. 8a is not linear due to its functionality (i.e., $y_{2} = y_{2} (\rho_{1} ,T,\kappa^{\prime\prime})$). However, a linear plot is observed when the data are depicted as $\ln ({{y_{2} } \mathord{\left/ {\vphantom {{y_{2} } {\left( {1 - \kappa^{\prime\prime}y_{2} } \right)}}} \right. \kern-0pt} {\left( {1 - \kappa^{\prime\prime}y_{2} } \right)}} - (\kappa^{\prime\prime} - 1)\ln \left( {{P \mathord{\left/ {\vphantom {P {P^{ * } }}} \right. \kern-0pt} {P^{ * } }}} \right) - {{A_{6} } \mathord{\left/ {\vphantom {{A_{6} } {T - B_{6} \ln \left( T \right) - D_{6} }}} \right. \kern-0pt} {T - B_{6} \ln \left( T \right) - D_{6} }}$ vs. ${{\rho_{1} } \mathord{\left/ {\vphantom {{\rho_{1} } {kg \cdot m^{ - 3} }}} \right. \kern-0pt} {kg \cdot m^{ - 3} }}$(Fig. 8b),which confirms the applicability of the new association model to the solubility data. Solubility of solids substances in scCO_2_ are best understood in terms of solvato-complex formation, thus the interactions between sitagliptin phosphate (solute) and supercritical carbon dioxide (solvent) is visualized as formation of a solvato-complex. The new association model (solvato-complex model) proposed in this study is able to capture solubility phenomena with least AARD% (i.e., 2.53%). Thus, present study confirms solvato-complex theory holds good for this sitagliptin phosphate–scCO_2_ system.

## Conclusion

Thesolubility of (R)-4-oxo-4-[3-(trifluoromethyl)-5,6-dihydro [1,2,4] triazolo[4,3-a] pyrazin-7(8H)-yl]-1-(2,4,5-trifluorophenyl) butan-2-amine (i.e., *sitagliptin phosphate*) was measured by a static method in the pressure range of 12–30 MPa for different temperature values (30, 318, 328 and 338 K). The measured solubilities range from (0.2074 to 0.698) × 10^–4^ mol fraction of (R)-4-oxo-4-[3-(trifluoromethyl)-5,6-dihydro [1,2,4] triazolo[4,3-a] pyrazin-7(8H)-yl]-1-(2,4,5-trifluorophenyl) butan-2-amine. Further, measured solubilities are reasonably correlated with the Chrastil model, the reformulated Chrastil model,the Méndez-Santiago and Teja (MST) model, the Bartle et al., model, the Kumar and Johnston (KJ) model. The newly proposed association model was able to correlate the solubility data with the lowest absolute relative deviation (2.53%). Calculated sublimation and solvation enthalpies of *Sitagliptin phosphate* in scCO_2_ are 40.416 kJ mol^−1^, − 24.535 kJ mol^−1^ for Bartle et al., model and Bartle et al., model + Chrastil model combination, respectively.

## List of symbols

*A*_*1*_*,B*_*1*_: Eq. (6) Constants
*A*_*2*_*, B*_*2*_: Eq. (7) Constants
*A*_*3*_*, B*_*3,*_* C*_*3*_: Eq. (8) Constants
*A*_*4*_*, B*_*4,*_* C*_*4*_: Eq. (9) Constants
*A*_*5*_*, B*_*5,*_* C*_*5*_: Eq. (10) Constants
*A*_*6*_*, B*_*6,*_* C*_*6*_*,D*_*6*_*,E*_*6*_*,F*_*6*_: Eq. (30) Constants
AARD%: Average absolute relative deviation percentage
$\hat{f}$: Fugacity in mixtuer
H_sol_: Solvation enthalpy(kJ/mol)
H_sub_: Sublimation enthalpy(kJ/mol)
H_total_: Total enthalpy(kJ/mol)
$$K_{f} ,$$: Equilibrium constant
M_CO2_: Molar mass of CO_2_ and drug (g/mol)
${M}_{solute}$: Solute Molar mass
P: System pressure
P^*^: Reference pressure
P^s^: Sublimation pressure(Pa)
*R*: Universal gas constant, 8.314 J/(molK)
scCO2: Supercritical carbon dioxide
T: System temperature (K)
${y}_{2}$: Drug solute solubility in mole fraction

## Greek symbols

$$\hat{\phi }$$: Fugacity coefficient
$$\beta ,\gamma ,\Delta \delta$$: Eq. (26) Constants
$$\rho_{1}$$: Solvent density
$$\kappa ,\kappa^ {\prime},\kappa^ {\prime\prime}$$: Association numbers
